# Lipoma Arborescens Where MRI Is a Boon

**DOI:** 10.7759/cureus.39212

**Published:** 2023-05-19

**Authors:** Nikita Bora, Pratap Parihar, Nishant Raj, Neha Shetty, Bhagyasri Nunna

**Affiliations:** 1 Radiodiagnosis, Jawaharlal Nehru Medical College, Datta Meghe Institute of Higher Education and Research, Wardha, IND

**Keywords:** mri, synovium, arborescens, lipoma, knee lesion, benign

## Abstract

Lipoma arborescens is a slow-progressing intra-articular benign lesion that typically affects the knee joint's suprapatellar recess. It occurs due to lipomatous proliferation of the synovium, giving a characteristic frond-like appearance. It is a rare cause of intermittent knee pain and joint effusion. We draw attention to this rare condition to increase the knowledge of its clinical symptoms and imaging characteristics, allowing for an early diagnosis and appropriate management. Magnetic resonance imaging (MRI) is considered the initial and the single imaging modality to evaluate this condition in the current era.

## Introduction

Lipoma arborescens is a rare idiopathic, intra-articular, non-neoplastic lesion of the synovium characterized by villous-lipomatous synovial proliferation and extensive sub-synovial tissue replacement by mature adipose tissue. Arzimanoglu first described it in 1957 [1]. Typically, it involves a unilateral diarthrodial joint. Although the knee is affected the most, other joints such as the hip, shoulder, elbow, and ankle have also been affected in a few. Although it is considered idiopathic, the association of pre-existing inflammatory processes of the joint is being considered. Chronic mechanical and inflammatory insult to the synovium may result in this rare proliferative condition. It shows frequent associations with inflammatory conditions, such as osteoarthritis and rheumatoid arthritis, in patients with high HbA1c or following long-standing knee injury. Patients usually present with chronic swelling, on-and-off pain, and effusions of the joint. With increasing effusion and pain, restriction of the range of motion can develop. Rarely can patients sometimes present with recurrent attacks of hemarthrosis and associated anemia [1]. With the ever-increasing utilization of magnetic resonance imaging (MRI), lipoma arborescens is being increasingly recognized. MRI is the only imaging modality because of its excellent contrast resolution and high sensitivity for fatty tissue. Pathognomonic MRI features include frond-like synovial projections appearing hyperintense on T1WI/T2WI in the pre-femoral region, showing fat suppression on short tau inversion recovery (STIR) sequence. Diagnosing lipoma arborescens was highly challenging until the advent of MRI. There had been only 13 cases documented in the literature till the mid-1990s. The conventional radiography results and the ambiguous clinical features necessitated surgical interventions and histopathology in the past [2].

Here, we present the clinical characteristics and imaging features of lipoma arborescens on plain radiograph, ultrasonography (USG), MRI, and histopathological examination of the postoperative specimen with particular emphasis on the role of MRI, which is now thought to be the best and the only imaging modality for evaluating intra-articular masses.

## Case presentation

Presentation and examination

A 68-year-old female patient, a retired schoolteacher, presented with complaints of pain and swelling in her right knee for six months. The pain was insidious in onset and on and off, with no aggravating and relieving factors. She has a history of trivial trauma to the knee two months back following which the pain has increased. There was no history of chronic systemic illness such as tuberculosis (TB), hypertension, and diabetes mellitus. There was no past significant surgical/operative history and no illness or malignancy in the family. Her laboratory results were unremarkable for any inflammatory changes and negative for rheumatoid factors. She has no history of similar illness in the past with no history of other joint involvement. On clinical examination, there was mild swelling of the right knee with crepitus felt on flexion and extension of the knee joint. Tenderness was felt over the medial aspect of the knee joint. No local rise in temperature was noted. Anterior and posterior drawer tests were unremarkable. McMurray's test was positive, suggesting the possibility of a medial meniscus tear.

Imaging findings

**Figure 1:**
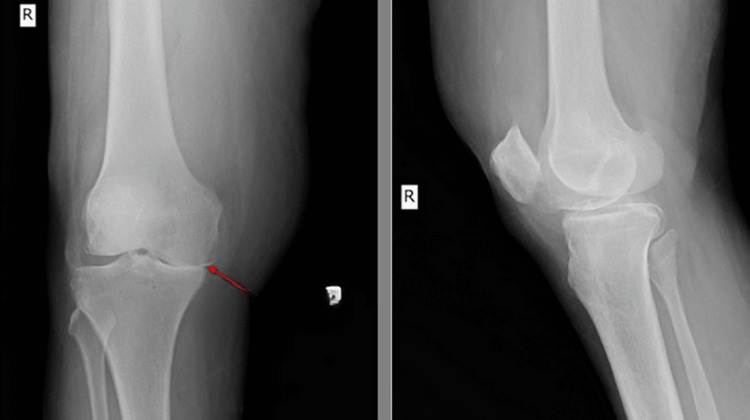
Right knee radiograph AP and lateral projections showing a reduction in joint space (medial>lateral) with osteophytes (red arrow) suggestive of osteoarthritic changes. AP: anteroposterior

She was advised to get an initial X-ray of the right knee (Figure 1), which showed a reduction in joint space (medial>lateral) with osteophytes suggestive of osteoarthritic changes. For further evaluation, she was advised to get an ultrasound of the right knee and an MRI. The patient, after that, underwent the mentioned investigation.

**Figure 2:**
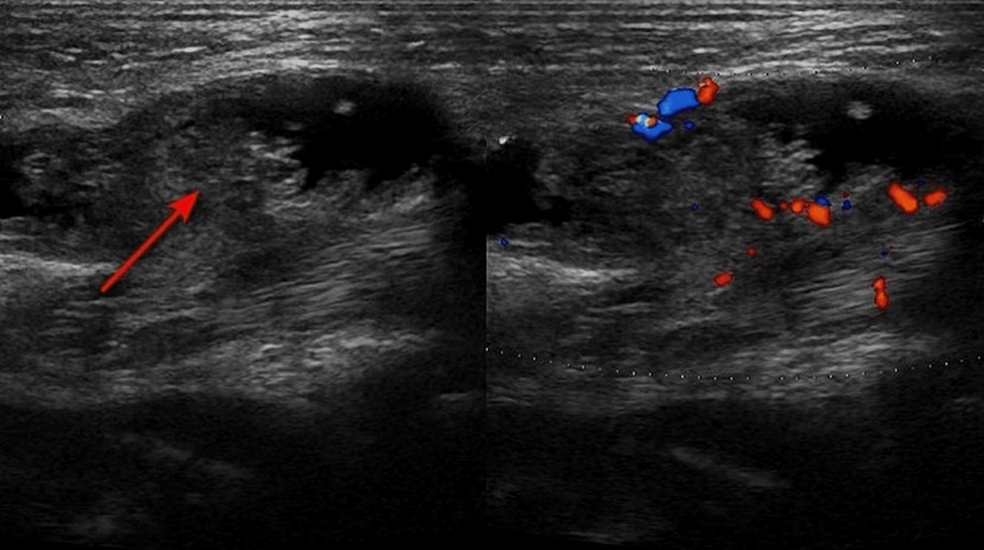
Longitudinal grayscale ultrasound image (left) and color Doppler image (right) of the right knee showing joint effusion with frond-like synovial proliferation in the suprapatellar region (red arrow) showing minimal to no vascularity in the synovial proliferation.

On USG (Figure 2), the right knee joint showed joint effusion and frond-like hyperechoic synovial proliferation projecting into the effusion in the suprapatellar region showing minimal to no vascularity on color Doppler.

**Figure 3:**
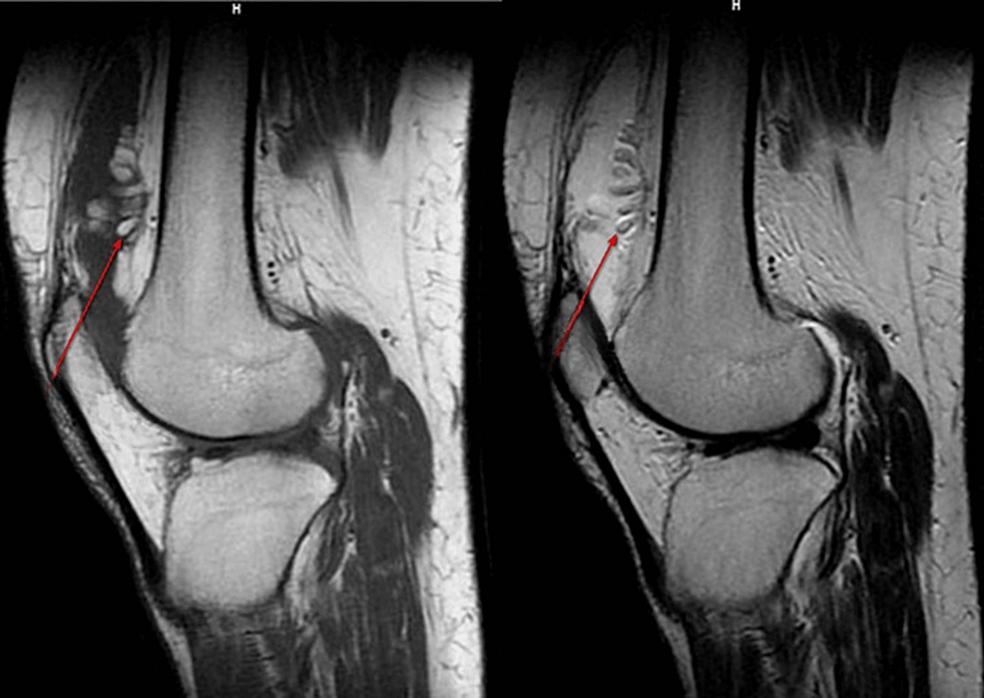
Sagittal T1WI (left) and T2WI (right) MRI showing frond-like projections (red arrow) in the pre-femoral region appearing hyperintense on both sequences. T1WI: T1-weighted image, T2WI: T2-weighted image, MRI: magnetic resonance imaging

**Figure 4:**
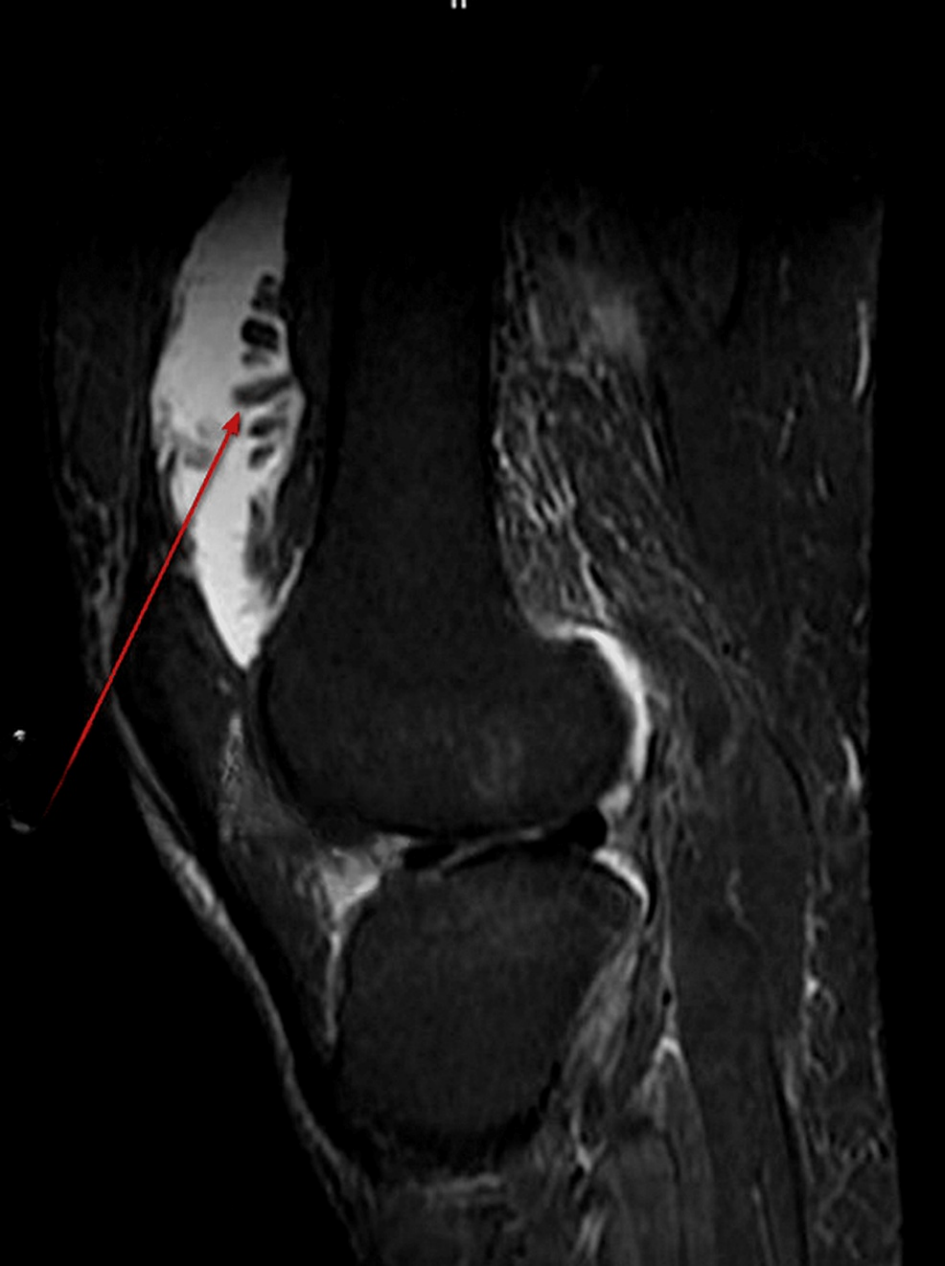
Sagittal STIR MRI showing fat suppression in frond-like projections (red arrow). STIR: short tau inversion recovery, MRI: magnetic resonance imaging

**Figure 5:**
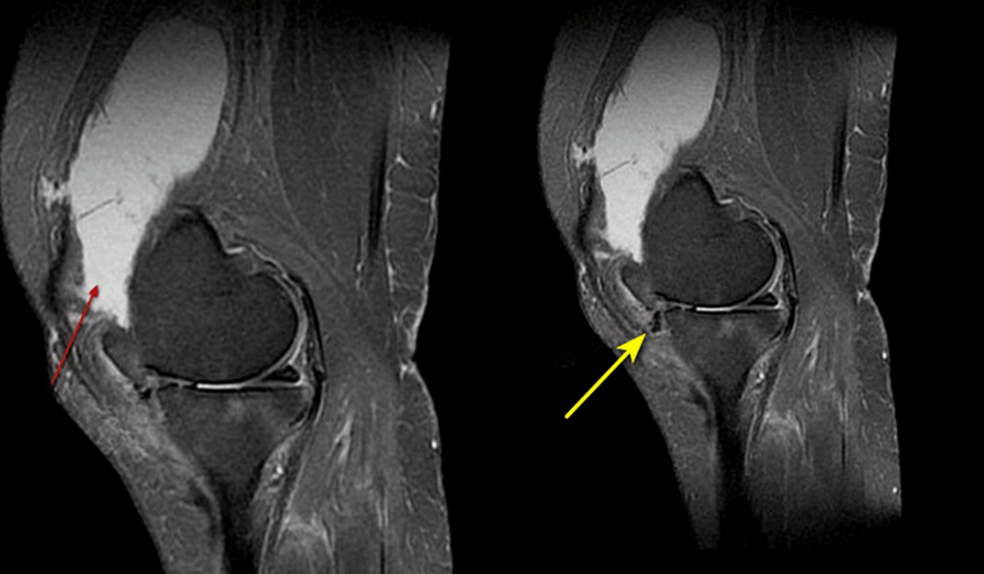
Sagittal PDFatSat MRI showing suprapatellar joint effusion (red arrow) and tear of the anterior horn of the medial meniscus (yellow arrow). PDFatSat: proton density fat saturation, MRI: magnetic resonance imaging

On MRI of the right knee, there was evidence of frond-like projections appearing hyperintense on T1WI/T2WI (Figure 3) in the pre-femoral region showing fat suppression on STIR (Figure 4) suggestive of lipoma arborescens. There was also evidence of intra-substance altered signal intensity in the medial meniscus with extrusion of the medial meniscus out of the joint space (Figure 5). Additionally, there was reduced joint space (medial>lateral) with osteophytes in the distal femur and proximal tibia, suggesting osteoarthritic changes. Also, fluid intensity collection is seen in the right suprapatellar bursa (Figure 5), pre-femoral bursa, and knee joint space with thickening of the synovium.

Treatment and follow-up

**Figure 6:**
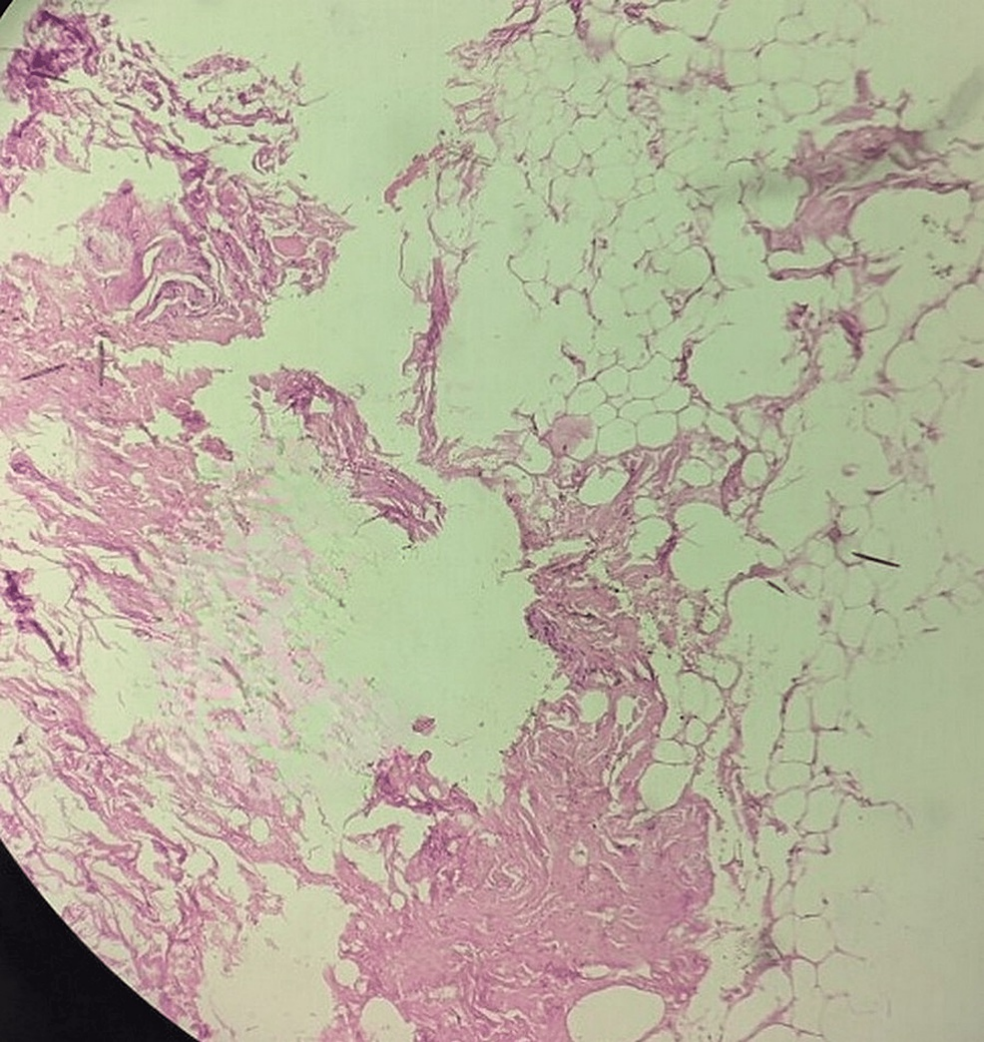
Hematoxylin and eosin stain on 40× magnification showing synovial lined villous proliferation wherein there was diffuse infiltration of fat cells.

The patient was subsequently managed conservatively with analgesics and anti-inflammatory medications and had been advised for total knee replacement for osteoarthritic and degenerative changes. The patient underwent a total knee replacement. The intra-operative specimen of the lesion was sent for histopathological examination. On histopathology (Figure 6), synovial lined villous proliferation was noted, wherein there was diffuse infiltration of fat cells.

## Discussion

Lipoma arborescens is a benign indolent condition. Minor sub-synovial fatty infiltration is prevalent, although such widespread lesions are rare. The average patient age is between the fifth and seventh decade; however, it can also affect young patients with persistent arthritis. One study initially reported that men were impacted more frequently than women, although another study with a larger sample size revealed no gender predilection. The disease often goes unnoticed, but misdiagnosing it with other complex lesions may lead to unnecessary interventions. Conventional radiography tends to miss such lesions because of indistinct features. Here, the role of MRI comes into play. MRI helps in early diagnosis by defining its nature, anatomical extensions, and associated local pathology. It gives a distinct signal on MRI, which sets it apart from other lesions that mimic it. As a result, it is thought that MRI is the best diagnostic method for its assessment.

On T1WI/T2WI, the frond-like projections of lipoma arborescence exhibit hyperintensity, which gets suppressed on fat saturation sequences. On T2WI or STIR sequences, the remaining non-fatty portion of the hypertrophied synovium exhibits heterogeneous high signal intensity. In contrast, on T1-weighted sequences, it exhibits intermediate to low signal intensity and no blooming on gradient imaging. Many present with underlying conditions such as joint effusion, which may be observed on MRI. Additionally, meniscal tears and degenerative changes are seen as pathological causes for the formation of lipoma arborescens. Differential diagnosis includes secondary osteochondromatosis from underlying severe osteoarthritis, pigmented villonodular synovitis, synovial hemangioma, intra-articular lipoma, and intra-articular liposarcoma [3]. The preferred treatment for this condition is open or arthroscopic synovectomy; however, underlying triggering factors must be addressed and treated accordingly. Arthroscopic synovectomy has always been preferred over open arthroscopic synovectomy, as there is early recovery and reduced hospital stay. Following surgery, the recurrence of the condition is negligible [4].

Tsifountoudis et al. [5] did a study on three patients with lipoma arborescens, highlighting its clinical and imaging features, with particular emphasis on the role of MRI in evaluating various differential diagnoses of lipoma arborescens. They concluded that MRI was the imaging modality of choice and described key pathognomonic characteristics. Their study observed the following imaging appearances: a large frond-like mass having a fat signal on all MRI sequences arising from synovium, the presence of a few fat-like globules with the extensive villous proliferation of synovium, and a mixed pattern. We observed a mixed pattern wherein a frond-like mass showed fat signal intensity on all MRI sequences with significant synovial proliferation.

De Vleeschhouwer et al. [6] described a case of a 31-year-old Dutch male with lipoma arborescens. They described clinical features, imaging features, and treatment options. In their case, they found MRI as the diagnostic modality of choice, with the appearance of multiple fatty synovial proliferation being pathognomonic for the diagnosis of lipoma arborescens. They also found osteoarthritic and degenerative changes as common associated imaging features. The imaging findings in their case were in congruence with our case findings.

## Conclusions

Lipoma arborescens is characterized by villous frond-like lipomatous proliferation of the synovium. It is a rare cause of intra-articular mass and frequently presents with joint swelling and indolent synovitis. Although it is considered to be idiopathic, it can occur as a response to chronic synovial irritation. Typically seen in patients with underlying chronic joint pathology, most often in the suprapatellar recess of the knee joint, other recess, joints, and tendon sheath can also be affected. It has got its distinct features on MRI. Many radiologists, rheumatologists, and orthopedic surgeons will encounter this in their practice and should not mistake it for malignant or other complex pathology as this may lead to unnecessary interventions. Synovectomy is the preferred surgical management; however, additional interventions may be needed if underlying pathological or triggering factors are associated with these conditions.

## References

[1] Spaans AJ, Turkenburg JL, Wagenmakers R (2013). Lipoma arborescens: an unusual cause of swelling of the knee. Radiol Case Rep.

[2] Coll JP, Ragsdale BD, Chow B, Daughters TC (2011). Best cases from the AFIP: lipoma arborescens of the knees in a patient with rheumatoid arthritis. Radiographics.

[3] (2023). Radsource: Lipoma arborescens. https://radsource.us/lipoma-arborescens.

[4] Sanamandra SK, Ong KO (2014). Lipoma arborescens. Singapore Med J.

[5] Tsifountoudis I, Kapoutsis D, Tzavellas AN, Kalaitzoglou I, Tsikes A, Gkouvas G (2017). Lipoma arborescens of the knee: report of three cases and review of the literature. Case Rep Med.

[6] De Vleeschhouwer M, Van Den Steen E, Vanderstraeten G, Huysse W, De Neve J, Vanden Bossche L (2016). Lipoma arborescens: review of an uncommon cause for swelling of the knee. Case Rep Orthop.

